# Prevalence and economic burden of dementia in the Arab world

**DOI:** 10.1192/bjo.2023.517

**Published:** 2023-07-13

**Authors:** Tarik Qassem, Lynn Itani, Walid Nasr, Dania Al-Ayyat, Syed Fahad Javaid, Hamed Al-Sinawi

**Affiliations:** Maudsley Health, Al-Amal Psychiatric Hospital, Dubai, UAE; Emirates Health Services, Al-Amal Psychiatric Hospital, Dubai, UAE; and Mohammed Bin Rashid University of Medicine and Health Sciences, Dubai, UAE; Maudsley Health, Al-Amal Psychiatric Hospital, Dubai, UAE; and Emirates Health Services, Al-Amal Psychiatric Hospital, Dubai, UAE; Emirates Health Services, Al-Amal Psychiatric Hospital, Dubai, UAE; Department of Psychiatry and Behavioral Sciences, United Arab Emirates University, Al Ain, UAE; Behavioral Medicine Department, Sultan Qaboos University Hospital, Muscat, Oman

**Keywords:** Dementia, Arab world, healthcare costs, economic burden, primary prevention

## Abstract

**Background:**

The growing prevalence of dementia is a global concern, especially in the Arab world, where updated economic impact data are scarce. Understanding its prevalence and cost is crucial for effective policies and support systems.

**Aims:**

To estimate dementia prevalence and cost in Arab countries for 2021.

**Method:**

United Nations population data and dementia prevalence estimates were used to calculate total cases. Direct costs were based on gross domestic product (GDP) per capita (purchasing power parity) and income classification. Indirect caregiver support costs were estimated using average monthly wages and two distinct scenarios.

**Results:**

The highest dementia prevalence among those aged more than 60 years was in Lebanon (4.88%), Tunisia (4.43%) and Algeria (4.19%). The total direct cost in the Arab region was $8.18 billion for those over 50 years old. Indirect costs ranged from $2.25 billion (best case) to $5.67 billion (worst case), with a mean value of $3.98 billion. Total dementia care costs (direct and indirect) under the mean scenario for the entire Arab world amounted to $12.17 billion, with costs as a percentage of GDP ranging from 0.05% (Sudan) to 0.44% (Lebanon).

**Conclusions:**

This study highlights dementia as a growing public health issue in the Arab world, with 1 329 729 individuals affected in 2021 and total costs between $10.43 billion and $13.90 billion. The findings emphasise the urgent need for investment in research and specialised services for older adults, particularly those with dementia.

Dementia is a clinical condition that is caused by neurodegeneration and results in cognitive decline and a reduced capacity for independent living.^1^ Individuals with dementia suffer from a range of symptoms including anxiety, irritability, low mood, hallucinations, delusions and sleep disorders.^2^ Alzheimer's disease is the most common type of dementia and accounts for around 60% of cases.^3,4^

Globally, it is estimated that around 55 million people were living with dementia in 2021, with numbers predicted to increase to 78 million in 2030^5^ and further to 152.8 million cases in 2050.^6^ Studies highlight geographical variations in the projected increases across countries and regions.^5,6^ The smallest percentage changes in numbers of projected dementia cases are expected in the high-income Asia Pacific (53%) and Western Europe (74%) regions. By contrast, the largest increases are anticipated in North Africa and the Middle East, including the Arab countries. The projected increases in cases can mainly be attributed to population growth and population ageing.

The costs of dementia are important to estimate as the condition incurs an economic burden on healthcare systems and caregivers. In 2009, Wimo and colleagues reported the worldwide cost of dementia as $422 billion, with $142 billion (34%) spent on informal care.^7^ A more recent study using a similar methodology estimated the cost of dementia in the Arab world in 2009 to range from $4.2 billion to $6.7 billion, with dementia costs accounting for over 0.75% of the total gross domestic product (GDP) in Mauritania, Iraq and Egypt, whereas the economic burden for the Gulf Cooperation Council was estimated at less than 0.25% of the GDP.^8^ A recent study by Wimo et al. found that the annual global societal costs of dementia in 2019 were US $1313.4 billion for 55.2 million people with dementia.^7^ These costs included direct medical costs (US $213.2 billion, 16%), direct social sector costs such as long-term care (US $448.7 billion, 34%), and informal care costs (US $651.4 billion, 50%). This research showed that although the majority of people with dementia live in low- and middle-income countries, the highest total and per-person costs are observed in high-income countries. Low- and middle-income countries accounted for 61.3% of people with dementia but only 26% of total costs, whereas high-income countries, with 38.7% of people with dementia, bore 74% of total costs. This discrepancy can be attributed to differences in healthcare systems, resource availability and accessibility, as well as varying costs of care across countries. This study did not examine individual countries but rather focused on categories of countries according to their income levels.^7^

Given the lack of updated estimates regarding the cost of dementia in the Arab world, the present work aims to calculate this economic burden for the year 2021 using the same methodology as that used by Wimo et al.^7–11^

## Method

The primary aim of this study was to calculate the prevalence and cost of dementia in Arab countries. To achieve this, we first needed to accurately estimate the number of individuals living with dementia in each Arab country. Our methodology, based on a model derived from previous studies,^7–11^ was as follows.

### Data collection

We collected population distribution and age-band data for both genders for all countries from the United Nations’ website.^12,13^ This foundational data allowed us to perform our calculations.

### Data extraction

We concentrated on the data specifically relevant to Arab countries and extracted the necessary information for our analysis.

### Prevalence estimates

Table 1 presents dementia prevalence estimates categorised by age bands and gender.^14^ This information was used to calculate the number of individuals living with dementia in each Arab country.

**Table 1:** Global dementia prevalence estimates according to age bands and across genders

	Dementia prevalence per 10 000	
Age band, years	Male	Female
50–59	12	33
60–69	153	195
70–79	431	554
80–89	1202	1611
90–99	2464	3879
100	5455	6694

This table shows dementia prevalence estimates according to age bands and across genders^14^, which we used to calculate the numbers of individuals with dementia in individual Arab countries.

### Application of prevalence estimates

We applied the prevalence figures from Table 1 to the population data for each Arab country. This enabled us to estimate the number of individuals living with dementia in each country, considering both the 10-year age bands and the gender distribution.

### Aggregation of dementia cases

We summed the estimated numbers of dementia cases across all age bands and genders to determine the total number of individuals living with dementia in each Arab country. To calculate the prevalence, we divided the numbers of individuals with dementia aged 50+ and 60+ years by the total populations aged 50+ and 60+ years for each Arab country.

### Cost calculation

#### Direct cost

The direct cost of dementia per person in each Arab country was calculated using GDP per capita, based on purchasing power parity (PPP) and the World Bank Income Classification. As depicted in Table 2, we collected GDP per capita PPP data for each Arab country and identified the World Bank Income Classification (low-income, lower-middle-income, upper-middle-income or high-income) from the most recent World Bank estimates.^15^

**Table 2:** GDP per capita PPP and income classification for Arab countries for the year 2021

Country	GDP per capita PPP (2021)	World Bank Income Classification
Algeria	12 128	Lower-middle-income
Bahrain	54 257	High-income
Comoros	3547	Lower-middle-income
Djibouti	5398	Lower-middle-income
Egypt	12 706	Lower-middle-income
Iraq	9846	Lower-middle-income
Jordan	10 133	Lower-middle-income
Kuwait	NA	High-income
Lebanon	14 257	Upper-middle-income
Libya	24 131	Upper-middle-income
Mauritania	5831	Lower-middle-income
Morocco	8853	Lower-middle-income
Oman	37 676	High-income
Qatar	102 018	High-income
Saudi Arabia	48 711	High-income
Somalia	1249	Low-income
State of Palestine	6199	Lower-middle-income
Sudan	4066	Low-income
Syria	NA	Low-income
Tunisia	11 282	Lower-middle-income
United Arab Emirates	76 609	High-income
Yemen	NA	Low-income

For countries with missing GDP per capita PPP data, values were obtained from the corresponding World Bank income group as follows:

- Kuwait (high-income, 2021): 48 894
- Syria (low-income, 2021): 2327
- Yemen (low-income, 2021): 2327.

We considered the cost per patient for the resources used in treating dementia in relation to the GDP per capita based on PPP. Wimo et al estimated that the direct cost per person with dementia is connected to the GDP per capita PPP.^7^ Their study also demonstrated that the direct costs of dementia vary among countries, depending on the World Bank income classification, as shown in Table 3.

**Table 3:** Direct cost of dementia in relation to country income

World Bank's income classification	Cost factor
Low-income	0.11
Lower-middle-income	0.17
Upper-middle-income	0.42
High-income	1.00

To estimate the direct cost per person with dementia in each Arab country, we multiplied the GDP per capita PPP by the corresponding factor based on income classification. By following this method, we were able to calculate the direct cost of dementia per person for each Arab country, taking into account the GDP per capita PPP and income classification. This resulted in an amount in United States Dollars (USD).

#### Indirect costs

To calculate indirect costs, the hourly wage of caregivers needs to be available. We extracted wage information for the year 2021 from data provided by the International Labour Organization (ILO), which includes the average monthly earnings in USD for various countries in the year 2021.^16^ Most Arab countries had no available data, apart from Comoros ($253.25), Egypt ($186) and Jordan ($475). For countries with missing data, the average wage was determined using calculated average monthly wages from the ILO data-set for the respective country category according to the World Bank. Table 4 depicts the calculated average monthly wages by World Bank income classification in 2021 (USD).

**Table 4:** Calculated average monthly wages by World Bank income classification in 2021 (USD)

World Bank income classification	Average monthly wage in 2021 (USD)
High-income	3640
Upper-middle-income	491
Lower-middle-income	269
Low-income	96

Numbers of dementia cases among individuals aged 50 years and above, along with monthly wages for each country, were collected from ILO and calculated or estimated as described before. This information was used to calculate the hourly wages in USD.

The cost of dementia was calculated under two different scenarios, as follows.

- Best case: in this scenario, it is assumed that caregivers spend 1.6 h per day providing care to individuals with dementia living in the community. The total cost is calculated by multiplying the hourly wage, the number of individuals with dementia living in the community (90% of the dementia population) and the number of hours spent per day by caregivers, for 365 days.
- Worst case: in this scenario, it is assumed that caregivers spend 3.7 h per day providing care to individuals with dementia living in the community. The total cost is calculated by multiplying the hourly wage, the number of individuals with dementia living in the community (which accounts for 99% of the dementia population, owing to cultural traditions in most Arab countries, where people with dementia are often cared for by informal caregivers such as spouses or offspring within their own household) and the higher number of hours spent per day by caregivers, for 365 days. The hourly wage of a retired caregiver was considered to be equivalent to the hourly wage of a caregiver of working age; although this strategy was not ideal, it was consistent with previous research on the burden of dementia.^8^

Data processing and visualisation were performed using a range of Python libraries and software. The core data manipulation and analysis tasks were carried out using pandas 1.4.4^17^ and NumPy 1.21.5.^18^ For data visualisation, the matplotlib library 3.5.2^19^ was employed. Geospatial data visualisation was achieved using Cartopy 0.21.1.^20^ All of these libraries were used within the Python 3.9 programming environment.^21^

## Results

**Fig. 1:**
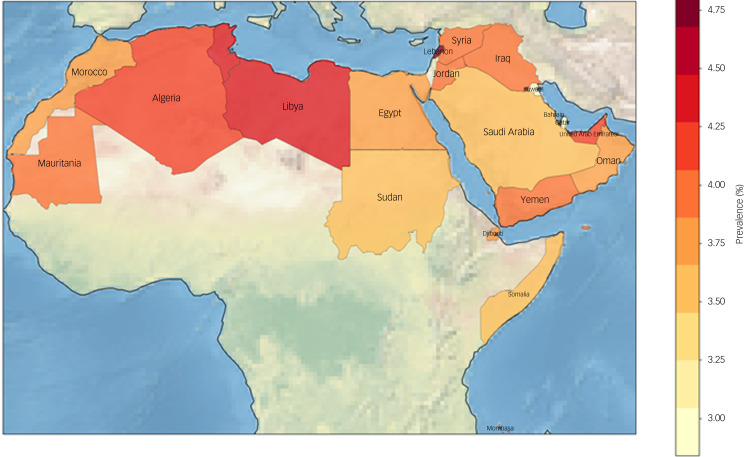
Prevalence of dementia in the Arab world. The map reports the prevalence of dementia in the Arab world in the population over the age of 60 years.

Table 5 shows the prevalence of dementia in the Arab world in the population aged 50 and 60 years or older. Figure 1 shows the prevalence of dementia in those aged over 60 years on a map of the Arab world.

**Table 5:** Prevalence of dementia in the Arab world

Country	In those aged 50+ years (%)	In those aged 60+ years (%)
Algeria	2.23	4.19
Bahrain	1.49	3.47
Comoros	2.13	4.03
Djibouti	1.94	3.84
Egypt	1.96	3.78
Iraq	1.86	3.93
Jordan	1.80	3.86
Kuwait	1.19	2.99
Lebanon	2.83	4.88
Libya	2.06	4.40
Mauritania	2.02	3.92
Morocco	2.16	3.84
Oman	1.74	3.84
Qatar	0.91	2.84
Saudi Arabia	1.32	3.65
Somalia	1.85	3.57
State of Palestine	2.08	4.01
Sudan	1.93	3.47
Syria	2.09	4.05
Tunisia	2.50	4.43
United Arab Emirates	1.33	4.09
Yemen	2.02	3.98

Supplementary Tables 1 and 2, available at https://doi.org/10.1192/bjo.2023.517, depict the total population and dementia figures for the countries belonging to the Arab world. The highest estimates of those living with dementia aged of 50 and 60 years or older were for Egypt (333 950 and 314 359), Algeria (182 966 and 173 890) and Morocco (172 417 and 164 015). The total direct cost of dementia in the region was estimated at $8184.9 million (range: $1.4– $3475.2 million) for those above 50 years of age. Table 6 shows estimates of direct costs attributed to dementia.

**Table 6:** Direct costs of dementia in individual Arab countries

Country	Direct cost (USD)
Algeria	377 240 533
Bahrain	192 014 714
Comoros	1 359 682
Djibouti	2 909 726
Egypt	721 361 640
Iraq	154 559 516
Jordan	48 301 342
Kuwait	583 896 232
Lebanon	236 991 519
Libya	239 813 152
Mauritania	9 785 510
Morocco	259 486 790
Oman	313 351 769
Qatar	274 734 786
Saudi Arabia	3 475 233 851
Somalia	3 710 484
State of Palestine	12 497 225
Sudan	41 138 732
Syria	16 786 628
Tunisia	145 451 309
United Arab Emirates	1 059 505 208
Yemen	14 726 594
Arab world	8 184 856 942

Table 7 presents the indirect costs of dementia in individual Arab countries, considering best- and worst-case scenarios for the percentage of individuals with dementia living in the community. Hourly wages are given in USD, and indirect costs are shown for both scenarios; the mean value is also given. The highest indirect cost in the best-case scenario was seen for Saudi Arabia ($853 139 364), whereas the lowest was for Comoros ($1 875 531). In the worst-case scenario, Saudi Arabia still had the highest indirect cost ($2 170 176 331), and Somalia had the lowest ($21 667 422). The highest hourly wages were found for Bahrain, Kuwait, Oman, Qatar and the United Arab Emirates ($22.75), whereas the lowest were for Somalia and Syria ($0.60). Overall, the total indirect cost for the Arab world ranged from $2 248 050 976 in the best-case scenario to $5 672 305 899 in the worst-case scenario, with a mean value of $3 983 257 396. Table 8 presents the total dementia care costs, both direct and indirect, in Arab countries, under best, worst and mean scenarios. Costs are shown in USD.

**Table 7:** Indirect costs of dementia in individual Arab countries

Country	Percentage of individuals with dementia living in the community		Hourly wage (USD)	Indirect cost in USD		
	Best-case scenario: 90%	Worst-case scenario: 99%		Best-case scenario	Worst-case scenario	Mean value
Algeria	164 669	181 136	1.68	161 630 170	411 146 974	286 388 572
Bahrain	3185	3503	22.75	42 318 816	107 633 126	74 975 971
Comoros	2029	2232	1.58	1 875 531	4 771 097	3 323 314
Djibouti	2853	3139	1.68	2 800 350	7 124 979	4 962 664
Egypt	300 555	330 610	1.16	204 229 627	519 508 328	361 868 977
Iraq	83 105	91 415	1.68	81 571 366	207 496 028	144 533 697
Jordan	25 236	27 760	2.97	43 722 211	111 219 977	77 471 094
Kuwait	10 747	11 822	22.75	142 794 448	363 242 596	253 018 522
Lebanon	35 621	39 183	3.07	63 892 348	162 525 747	113 209 047
Libya	21 295	23 425	3.07	38 196 220	97 163 709	67 679 964
Mauritania	8884	9773	1.68	8 720 053	22 182 997	15 451 525
Morocco	155 175	170 692	1.68	152 311 374	387 440 924	269 876 149
Oman	7485	8233	22.75	99 452 540	252 967 035	176 209 787
Qatar	2423	2666	22.75	32 194 188	81 915 476	57 054 832
Saudi Arabia	64 209	70 630	22.75	853 139 364	2 170 176 331	1 511 657 847
Somalia	24 309	26 740	0.60	8 517 873	21 667 422	15 092 647
State of Palestine	10 672	11 739	1.68	10 475 057	26 645 472	18 560 264
Sudan	82 782	91 060	0.60	29 006 812	73 785 918	51 396 365
Syria	59 030	64 933	0.60	20 684 112	52 615 209	36 649 660
Tunisia	68 250	75 075	1.68	66 990 503	170 407 092	118 698 797
United Arab Emirates	12 447	13 691	22.75	165 382 199	420 669 462	293 025 830
Yemen	51 786	56 964	0.60	18 145 814	46 157 92	32 151 871
Arab world	1 196 747	1 316 421	163	2 248 050 976	5 672 305 899	3 983 257 396

**Table 8:** Total dementia care costs (direct and indirect) in Arab countries: best, worst and mean scenarios

Country	Total cost in USD		
	Best-case scenario	Worst-case scenario	Mean value
Algeria	538 870 703	788 387 507	663 629 105
Bahrain	234 333 530	299 647 840	266 990 685
Comoros	3 235 213	6 130 779	4 682 996
Djibouti	5 710 076	10 034 705	7 872 390
Egypt	925 591 267	1 240 869 968	1 083 230 617
Iraq	236 130 882	362 055 544	299 093 213
Jordan	92 023 553	159 521 319	125 772 436
Kuwait	726 690 680	947 138 828	836 914 754
Lebanon	300 883 867	399 517 266	350 200 566
Libya	278 009 372	336 976 861	307 493 116
Mauritania	18 505 563	31 968 507	25 237 035
Morocco	411 798 164	646 927 714	529 362 939
Oman	412 804 309	566 318 804	489 561 556
Qatar	306 928 974	356 650 262	331 789 618
Saudi Arabia	4 328 373 215	5 645 410 182	4 986 891 698
Somalia	12 228 357	25 377 906	18 803 131
State of Palestine	22 972 282	39 142 697	31 057 489
Sudan	70 145 544	114 924 650	92 535 097
Syria	37 470 740	69 401 837	53 436 288
Tunisia	212 441 812	315 858 401	264 150 106
United Arab Emirates	1 224 887 407	1 480 174 670	1 352 531 038
Yemen	32 872 408	60 884 523	46 878 465
Arab world	10 432 907 918	13 903 320 770	12 168 114 344

**Fig. 2:**
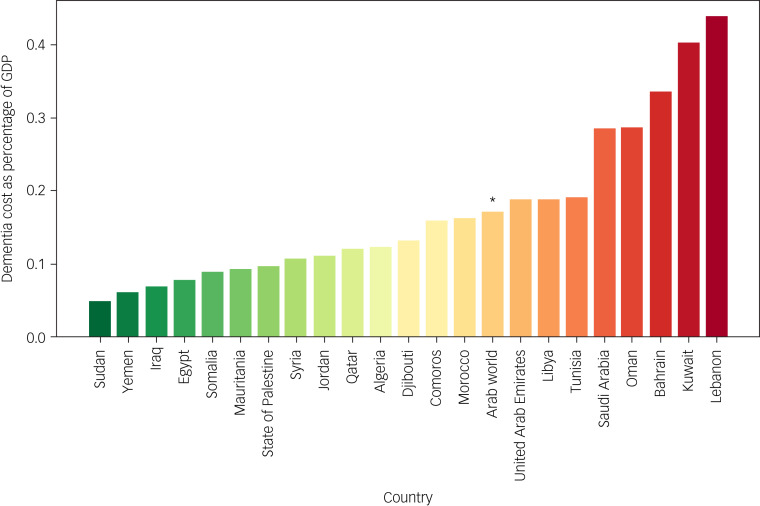
Total cost of dementia as a percentage of GDP in the Arab world countries. The asterisk denotes the total cost of dementia as a percentage of GDP in the Arab world as a whole.

The figures reveal considerable variation in costs across the Arab countries. Saudi Arabia incurred the highest mean dementia care costs, amounting to $4 986 891 698. By contrast, Comoros had the lowest mean cost at $4 682 996. Other countries, including the United Arab Emirates, Egypt and Algeria, also had comparatively high mean costs, exceeding $1 billion, $1 billion and $663 million, respectively. At the other end of the spectrum, countries including Djibouti, Somalia and the State of Palestine had relatively low mean costs, ranging between $7 872 390 and $31 057 489. Further, the combined mean dementia care cost for the entire Arab world was $12 168 114 344. Supplementary Table 3 depicts the ratio between the indirect and total cost of dementia in Arab countries. Table 9 and Fig. 2 depict the total cost of dementia as a percentage of GDP for various countries in the Arab world, illustrating the economic burden of dementia in each country. Notably, the percentages ranged from a low of 0.05% in Sudan to a high of 0.44% in Lebanon. In addition, some countries, including Kuwait (0.40%) and Bahrain (0.34%), exhibited higher percentages of dementia costs relative to GDP compared with others in the region. The table also includes an average percentage for the entire Arab world, which was calculated to be 0.18%.

**Table 9:** Total cost of dementia as a percentage of GDP by country in the Arab world

Country	Total cost of dementia/GDP (%)
Algeria	0.12
Bahrain	0.34
Comoros	0.16
Djibouti	0.13
Egypt	0.08
Iraq	0.07
Jordan	0.11
Kuwait	0.40
Lebanon	0.44
Libya	0.19
Mauritania	0.09
Morocco	0.16
Oman	0.29
Qatar	0.12
Saudi Arabia	0.28
Somalia	0.09
State of Palestine	0.10
Sudan	0.05
Syria	0.11
Tunisia	0.19
United Arab Emirates	0.19
Yemen	0.06
Arab world	0.18

## Discussion

The prevalence of dementia in the Arab world has become an increasingly important public health issue, as evidenced by the growing number of affected individuals and the associated economic burden on societies. This paper seeks to emphasise the significant issue of dementia in the Arab world, with the goal of attracting the attention of decision makers to invest in research and specialised services for older adults, particularly those affected by dementia. The choice of this topic was based on the scarcity of research focusing on dementia in the Arab world. A systematic review by El-Metwally et al found only 18 studies that investigated the prevalence of dementia in the region, with only six of them being community based.^22–28^ A 2017 review of dementia prevalence studies in Egypt found that dementia prevalence ranged from 2.01% to 5.07%.^29^ One study from the upper desert areas of Egypt reported that the total prevalence of Alzheimer's dementia was 1% among the population aged 50 years and more, reaching 2.9% at 70–79 years.^30^

Although the main focus of this study was the prevalence of dementia, encompassing both diagnosed and undiagnosed cases, it is worth briefly mentioning the challenge of undiagnosed dementia. As underscored in the *World Alzheimer Report 2021: Journey through the Diagnosis of Dementia*, obtaining a diagnosis remains a significant issue for people living with dementia; Alzheimer's Disease International (ADI) estimates that globally, 75% of people with dementia are not diagnosed, with that percentage believed to be as high as 90% in some lower- and middle-income countries.^5^ Despite the limited data and the focus of the ADI report on diagnosis rates rather than dementia prevalence, the authors anticipate a considerably high percentage of undiagnosed cases in the Arab world.

According to our estimates, there were 1 329 729 individuals with dementia in the Arab world in 2021. The prevalence of dementia varied across countries, with the highest rates observed in Tunisia (4.20%) and the lowest rates in Qatar (1.57%). The prevalence of dementia in the Arab world was 2.16% in those aged 50+ years and 3.84% in those aged 60+ years. The highest estimates of those living with dementia above the age of 50 and 60 years were in Egypt (312 399), Algeria (169 784) and Morocco (159 890), whereas Comoros had the lowest number (1166). There were differences in dementia prevalence rankings between the 50+ and 60+ age groups across different countries. For instance, the United Arab Emirates experienced a significant increase in ranking, moving from fourth place with a 1.33% prevalence in the 50+ age group to 18th place with a 4.09% prevalence in the 60+ age group. Conversely, Morocco saw a considerable decrease in ranking. However, some countries, including Qatar, Kuwait, Tunisia, Syria and Lebanon, showed no difference in their rankings between the two age groups. These variations in dementia prevalence rankings between age groups can be attributed to factors such as population age distribution, dementia risk factors, access to healthcare and support services, and cultural and lifestyle factors. In this study, the differences in ranking were probably due to demographics, specifically the differences in population age distribution between the countries. A larger proportion of older adults in the 60+ age group could lead to a substantial increase in dementia prevalence, owing to the increased risk of dementia with age, whereas countries with a younger population might have a lower prevalence, resulting in a smaller change in ranking.

This study found an economic burden of dementia for those aged 50 years and above amounting to a total of 10.43 billion USD in the best-case scenario and 13.90 billion USD in the worst-case scenario for the year 2021. It is important to note that these costs are estimates rather than precise calculations. Using a comparable methodology, the cost estimates for the year 2009 in the Arab region were found to be 4.212 billion USD for the best-case scenario and 6.707 billion USD for the worst-case scenario. There are several reasons for this significant increase in the economic burden of dementia, including demographic changes; for instance, the total population above the age of 60 in 2021 was about 2.5 times that in 2009. The correlation between GDP per capita and direct cost has also been updated, and an increase in the average GDP per capita estimate for the region was found. The direct costs of dementia care in Arab countries are influenced by each country's GDP per capita and income classification, with higher costs observed in high-income countries such as Saudi Arabia (2039 USD per capita) and the United Arab Emirates (2371 USD per capita). By contrast, lower costs were seen in lower-middle-income countries such as Yemen (223 USD per capita) and Djibouti (256 USD per capita).

Indirect costs, which include loss of productivity due to caregiving, can also significantly contribute to the overall economic burden of dementia. These costs are typically higher in countries with larger proportions of informal care provision by family members or friends. For instance, informal caregiving costs were found to be exceptionally high in certain countries, including Egypt, Algeria and Morocco; by contrast, the direct costs, which are correlated with GDP per capita, were much lower in those countries. These observations probably reflect the prevailing structure of care services, which tend to rely heavily on informal rather than formal caregiving arrangements.

As this study examined dementia prevalence in 2021, when the COVID-19 pandemic was raging, it is essential to acknowledge the potential influence of the pandemic on dementia care and its costs. One cannot attempt to ignore the devastating impact of the COVID-19 pandemic on humanity during 2020 and 2021. This global crisis has strained healthcare systems and posed significant challenges for most individuals, especially those living with dementia and their caregivers. Factors such as heightened social isolation, disrupted healthcare services and increased stress levels may contribute to the exacerbation of dementia symptoms and increase the risk of developing the condition. Furthermore, COVID-19 infections have been linked to higher risk of cognitive decline, particularly among older adults.^31,32^

Matias-Guiu et al found that living in care homes was the most significant factor for increased risk of infection and death among dementia patients.^33^ This finding suggests that the pandemic may have disproportionately affected individuals with dementia in care facilities, which could have implications for the costs of dementia care. Cascini et al^34^ assessed the incidence of SARS-CoV-2 infection and COVID-19-related death among dementia patients, identifying risk factors for both infection and mortality. Whereas this discussion paper does not directly investigate the relationship between COVID-19 and dementia prevalence or care in the Arab world, future research should explore the influence of the pandemic on the overall dementia landscape in the region, taking into account its potential impact on the cost of dementia care.^33,34^

The present study had some limitations, including its reliance on secondary data sources for population, prevalence and cost estimates. This may have introduce some inaccuracies in our calculations, as the quality and completeness of the data varied among countries. In addition, the cost estimates may not fully capture the complexity of dementia care provision in each country, as they were based on GDP per capita and income classification rather than detailed cost data from individual countries. This analysis focused on the Arab world, and our findings may not be generalisable to other regions with different demographic, economic and healthcare characteristics owing to economic or political instability in some of the Arab countries, potential bureaucratic or regulatory hurdles, and a lack of standardised neuropsychometric research instruments in the region.^35,36^ Another limitation to consider is the potential influence of the COVID-19 pandemic on dementia care and its costs during the study period, which examined dementia prevalence in 2021 when the pandemic was still ongoing. The pandemic has strained healthcare systems and introduced additional challenges for individuals living with dementia and their caregivers, as discussed earlier. Factors such as heightened social isolation, disrupted healthcare services and increased stress levels could have affected dementia symptoms and risks, and such effect may not have been fully captured in our analysis. Moreover, the implications of the pandemic for the cost of dementia care, as suggested by Matias-Guiu et al and Cascini et al,^33,34^ might not be adequately reflected in our estimates. Future research should further investigate the influence of the pandemic on the overall dementia landscape in the Arab world, considering its potential impact on care costs as a factor within the study's limitations.

Despite these limitations, we hope this study will provide useful insights into the prevalence and costs of dementia in Arab countries. These findings could help to inform policymakers, healthcare providers and researchers in their efforts to address the challenges posed by dementia in the region. As the population in Arab countries continues to age and the prevalence of dementia risk factors increases, it is crucial to invest in prevention, early detection and support services to reduce the impact of dementia on individuals, families and society. To tackle the growing issue of dementia in the Arab world, we make the following recommendations. (a) Enhance investment in dementia research to increase understanding of regional prevalence, risk factors and cultural aspects. This will aid in creating targeted interventions and policies for dementia prevention and care. (b) Strengthen national dementia strategies by improving early diagnosis, offering integrated care services and fostering dementia-friendly communities. This will reduce stigma, enhance quality of life for people with dementia and support their caregivers. (c) Create and implement training programmes for healthcare professionals, particularly in primary care settings, to improve dementia diagnosis and management. In accordance with the World Alzheimer 2022 report, emphasise person-centred care, caregiver support, evolving clinical roles and dementia education. This will result in a stable, effective, and cost-efficient care economy for people with dementia and their families.^37^ (d) Develop specialised mental health services for older adults, focusing on early identification, prevention and intervention. Collaborate with multidisciplinary teams to provide comprehensive, integrated care promoting mental well-being and quality of life for older persons in the Arab world. (e) Promote collaboration between governments, researchers, non-governmental organisations and international organisations to share best practices, resources and data on dementia care and support. (f) Increase funding for public awareness campaigns to enhance dementia knowledge and encourage early detection and intervention, reducing the societal and economic impact of dementia in the Arab world. (g) Implement dementia-friendly environment design principles from the World Alzheimer Report 2020 to improve quality of life for people with dementia and help them to maintain their abilities.^38^

In conclusion, this paper highlights the significant issue of dementia in the Arab world and underscores the urgent need for action from decision makers to invest in research and specialised services for older adults, particularly those affected by dementia. With the projected increase in the prevalence of dementia in the coming decades, addressing this public health challenge is critical to ensuring the well-being of older adults and reducing the economic burden on societies in the region.

## Data Availability

The data-sets used and analysed during the current study have been cited and are available upon request. The data that support the findings of this study are available from the corresponding author, S.F.J., upon reasonable request.

## Supplementary material

For supplementary material accompanying this paper visit http://doi.org/10.1192/bjo.2023.517. click here to view supplementary material
