# Supplementary material for: Prevalence and economic burden of dementia in the Arab world
Source: BJPsych Open. 2023 Jul 13;9(4):e126. doi: 10.1192/bjo.2023.517 (PMC10375885; doi:10.1192/bjo.2023.517)
Supplement: Supplementary file 1 [file bjosup.zip › S2056472423005173sup001.docx]

**S1. Population aged 50+ and 60+ in Arab countries**

|  | **Population aged 50+** | | | **Population aged 60+** | | |
| --- | --- | --- | --- | --- | --- | --- |
| **Country** | **Female** | **Male** | **Total** | **Female** | **Male** | **Total** |
| **Algeria** | 4,124,067 | 4,092,054 | 8,216,121 | 2,126,174 | 2,023,232 | 4,149,406 |
| **Bahrain** | 94,325 | 143,469 | 237,794 | 41,329 | 52,493 | 93,822 |
| **Comoros** | 53,553 | 52,138 | 105,691 | 28,083 | 24,989 | 53,072 |
| **Djibouti** | 86,436 | 76,799 | 163,235 | 41,998 | 35,414 | 77,412 |
| **Egypt** | 8,989,964 | 8,040,395 | 17,030,359 | 4,640,521 | 3,676,425 | 8,316,946 |
| **Iraq** | 2,744,068 | 2,231,416 | 4,975,484 | 1,268,296 | 915,174 | 2,183,470 |
| **Jordan** | 754,269 | 802,879 | 1,557,148 | 343,173 | 334,426 | 677,599 |
| **Kuwait** | 330,670 | 671,539 | 1,002,209 | 120,173 | 238,968 | 359,141 |
| **Lebanon** | 759,992 | 640,438 | 1,400,430 | 432,688 | 349,156 | 781,844 |
| **Libya** | 596,484 | 553,316 | 1,149,800 | 270,479 | 234,627 | 505,106 |
| **Mauritania** | 256,602 | 231,082 | 487,684 | 124,482 | 112,593 | 237,075 |
| **Morocco** | 4,081,304 | 3,913,350 | 7,994,654 | 2,209,708 | 2,058,543 | 4,268,251 |
| **Oman** | 193,066 | 283,665 | 476,731 | 98,205 | 104,688 | 202,893 |
| **Qatar** | 75,027 | 220,058 | 295,085 | 26,021 | 56,163 | 82,184 |
| **Saudi Arabia** | 1,823,241 | 3,564,580 | 5,387,821 | 738,881 | 1,033,118 | 1,771,999 |
| **Somalia** | 778,220 | 684,984 | 1,463,204 | 385,455 | 323,611 | 709,066 |
| **State of Palestine** | 301,266 | 267,978 | 569,244 | 151,642 | 127,644 | 279,286 |
| **Sudan** | 2,634,549 | 2,143,498 | 4,778,047 | 1,361,613 | 1,129,714 | 2,491,327 |
| **Syria** | 1,697,776 | 1,447,210 | 3,144,986 | 851,960 | 676,654 | 1,528,614 |
| **Tunisia** | 1,588,434 | 1,449,974 | 3,038,408 | 873,408 | 765,706 | 1,639,114 |
| **United Arab Emirates** | 308,380 | 730,444 | 1,038,824 | 122,665 | 184,549 | 307,214 |
| **Yemen** | 1,520,782 | 1,331,379 | 2,852,161 | 757,458 | 602,020 | 1,359,478 |
| **Total** | 33,792,475 | 33,572,645 | 67,365,120 | 17,014,412 | 15,059,907 | 32,074,319 |
