# Supplementary material for: Prevalence and economic burden of dementia in the Arab world
Source: BJPsych Open. 2023 Jul 13;9(4):e126. doi: 10.1192/bjo.2023.517 (PMC10375885; doi:10.1192/bjo.2023.517)
Supplement: Supplementary file 1 [file bjosup.zip › S2056472423005173sup002.docx]

**S2. Number of individuals with dementia in Arab countries**

|  | **Number of individuals with dementia aged 50+** | | | **Number of individuals with dementia aged 60+** | | |
| --- | --- | --- | --- | --- | --- | --- |
| **Country** | **Females** | **Males** | **Total** | **Females** | **Males** | **Total** |
| **Algeria** | 110,865 | 72,101 | 182,966 | 104,272 | 69,618 | 173,890 |
| **Bahrain** | 1,961 | 1,578 | 3,539 | 1,786 | 1,468 | 3,254 |
| **Comoros** | 1,390 | 865 | 2,255 | 1,306 | 833 | 2,139 |
| **Djibouti** | 2,007 | 1,164 | 3,171 | 1,861 | 1,114 | 2,975 |
| **Egypt** | 221,913 | 112,037 | 333,950 | 207,559 | 106,800 | 314,359 |
| **Iraq** | 62,441 | 29,898 | 92,339 | 57,571 | 28,319 | 85,890 |
| **Jordan** | 17,229 | 10,812 | 28,041 | 15,873 | 10,250 | 26,123 |
| **Kuwait** | 5,400 | 6,542 | 11,942 | 4,705 | 6,022 | 10,727 |
| **Lebanon** | 26,530 | 13,049 | 39,579 | 25,450 | 12,700 | 38,150 |
| **Libya** | 15,004 | 8,658 | 23,662 | 13,928 | 8,276 | 22,204 |
| **Mauritania** | 6,044 | 3,828 | 9,872 | 5,608 | 3,686 | 9,294 |
| **Morocco** | 107,004 | 65,413 | 172,417 | 100,828 | 63,187 | 164,015 |
| **Oman** | 4,898 | 3,419 | 8,317 | 4,585 | 3,204 | 7,789 |
| **Qatar** | 1,180 | 1,513 | 2,693 | 1,018 | 1,317 | 2,335 |
| **Saudi Arabia** | 38,640 | 32,704 | 71,344 | 35,062 | 29,666 | 64,728 |
| **Somalia** | 17,076 | 9,935 | 27,011 | 15,780 | 9,502 | 25,282 |
| **State of Palestine** | 7,658 | 4,200 | 11,858 | 7,165 | 4,032 | 11,197 |
| **Sudan** | 57,037 | 34,943 | 91,980 | 52,836 | 33,726 | 86,562 |
| **Syria** | 43,132 | 22,457 | 65,589 | 40,340 | 21,532 | 61,872 |
| **Tunisia** | 48,505 | 27,329 | 75,834 | 46,146 | 26,508 | 72,654 |
| **United Arab Emirates** | 7,641 | 6,189 | 13,830 | 7,028 | 5,533 | 12,561 |
| **Yemen** | 37,550 | 19,990 | 57,540 | 35,031 | 19,115 | 54,146 |
| **Total** | 841,105 | 488,624 | 1,329,729 | 785,738 | 466,408 | 1,252,146 |
