# Supplementary material for: Prevalence and economic burden of dementia in the Arab world
Source: BJPsych Open. 2023 Jul 13;9(4):e126. doi: 10.1192/bjo.2023.517 (PMC10375885; doi:10.1192/bjo.2023.517)
Supplement: Supplementary file 1 [file bjosup.zip › S2056472423005173sup003.docx]

**S3. Ratio between the indirect and total cost of dementia**

| **Country** | **Indirect/total cost of dementia** |
| --- | --- |
| **Algeria** | 0.43 |
| **Bahrain** | 0.28 |
| **Comoros** | 0.71 |
| **Djibouti** | 0.63 |
| **Egypt** | 0.33 |
| **Iraq** | 0.48 |
| **Jordan** | 0.62 |
| **Kuwait** | 0.30 |
| **Lebanon** | 0.32 |
| **Libya** | 0.22 |
| **Mauritania** | 0.61 |
| **Morocco** | 0.51 |
| **Oman** | 0.36 |
| **Qatar** | 0.17 |
| **Saudi Arabia** | 0.30 |
| **Somalia** | 0.80 |
| **State of Palestine** | 0.60 |
| **Sudan** | 0.56 |
| **Syria** | 0.69 |
| **Tunisia** | 0.45 |
| **United Arab Emirates** | 0.22 |
| **Yemen** | 0.69 |
| **Arab World** | 0.33 |
